# Reduced-iodine-dose dual-energy coronary CT angiography: qualitative and quantitative comparison between virtual monochromatic and polychromatic CT images

**DOI:** 10.1007/s00330-021-07809-w

**Published:** 2021-03-19

**Authors:** David C. Rotzinger, Salim A. Si-Mohamed, Jérôme Yerly, Sara Boccalini, Fabio Becce, Loïc Boussel, Reto A. Meuli, Salah D. Qanadli, Philippe C. Douek

**Affiliations:** 1 Department of Diagnostic and Interventional Radiology, Division of Cardiothoracic and Vascular Imaging, Lausanne University Hospital (CHUV), Rue du Bugnon 46, 1011 Lausanne, Switzerland; 2 Faculty of Biology and Medicine (FBM), University of Lausanne (UNIL), Lausanne, Switzerland; 3 Radiology Department, Hospices Civils de Lyon, 59 Boulevard Pinel, 69500 Bron, France; 4 University Claude Bernard Lyon 1, CREATIS, CNRS UMR 5220, INSERM U1206, INSA-Lyon, Lyon, France; 5 Department of Diagnostic and Interventional Radiology, Center for Biomedical Imaging (CIBM), Lausanne University Hospital (CHUV), Lausanne, Switzerland; 6 Department of Diagnostic and Interventional Radiology, Lausanne University Hospital (CHUV), Rue du Bugnon 46, 1011 Lausanne, Switzerland

**Keywords:** Coronary vessels, Computed tomography angiography, Iodine, Phantoms, imaging, Dimensional measurement accuracy

## Abstract

**Objectives:**

To quantitatively evaluate the impact of virtual monochromatic images (VMI) on reduced-iodine-dose dual-energy coronary computed tomography angiography (CCTA) in terms of coronary lumen segmentation in vitro, and secondly to assess the image quality in vivo, compared with conventional CT obtained with regular iodine dose.

**Materials and methods:**

A phantom simulating regular and reduced iodine injection was used to determine the accuracy and precision of lumen area segmentation for various VMI energy levels. We retrospectively included 203 patients from December 2017 to August 2018 (mean age, 51.7 ± 16.8 years) who underwent CCTA using either standard (group A, *n* = 103) or reduced (group B, *n* = 100) iodine doses. Conventional images (group A) were qualitatively and quantitatively compared with 55-keV VMI (group B). We recorded the location of venous catheters.

**Results:**

In vitro, VMI outperformed conventional CT, with a segmentation accuracy of 0.998 vs. 1.684 mm^2^, respectively (*p* < 0.001), and a precision of 0.982 vs. 1.229 mm^2^, respectively (*p* < 0.001), in simulated overweight adult subjects. In vivo, the rate of diagnostic CCTA in groups A and B was 88.4% (*n* = 91/103) vs. 89% (*n* = 89/100), respectively, and noninferiority of protocol B was inferred. Contrast-to-noise ratios (CNR) of lumen versus fat and muscle were higher in group B (*p* < 0.001) and comparable for lumen versus calcium (*p* = 0.423). Venous catheters were more often placed on the forearm or hand in group B (*p* < 0.001).

**Conclusion:**

In vitro, low-keV VMI improve vessel area segmentation. In vivo, low-keV VMI allows for a 40% iodine dose and injection rate reduction while maintaining diagnostic image quality and improves the CNR between lumen versus fat and muscle.

**Key Points:**

*• Dual-energy coronary CT angiography is becoming increasingly available and might help improve patient management.*

*• Compared with regular-iodine-dose coronary CT angiography, reduced-iodine-dose dual-energy CT with low-keV monochromatic image reconstructions performed better in phantom-based vessel cross-sectional segmentation and proved to be noninferior in vivo.*

*• Patients receiving reduced-iodine-dose dual-energy coronary CT angiography often had the venous catheter placed on the forearm or wrist without compromising image quality.*

**Supplementary Information:**

The online version contains supplementary material available at 10.1007/s00330-021-07809-w.

## Introduction

Coronary computed tomography angiography (CCTA) has become the most widely used method for non-invasive assessment of coronary artery disease [1]. To minimize adverse effects from contrast medium (CM) injection, iodine dose reduction should be a continuing effort, especially for patients with impaired kidney function or cardiopulmonary decompensation [2], but also in general, since lower iodine usage can reduce CT-induced DNA damage [3] and save costs. Furthermore, using lower injection rates could help manage patients with poor vein integrity when the catheter is inserted in the forearm or the hand [4]. Then again, efforts to save iodinated CM should be implemented with care in clinical routine to maintain CT examinations’ diagnostic performance and ultimately patient outcomes.

Lately, dual-energy CT and virtual monochromatic images (VMI) have shown promise in reducing CM dose in phantoms [5] and small patient cohorts, using fast kV switching [6, 7] or dual-layer spectral detector [8, 9] technology. However, these initial studies failed to perform a sample size calculation and did not apply appropriate noninferiority statistics to prove the similarity between conventional CCTA and reduced-CM-dose dual-energy CCTA [10]. Furthermore, data regarding the impact of VMI on image quality, especially vessel diameter accuracy and precision, are still limited. Previous research has attempted to assess the accuracy and precision of vessel lumen area measurements with conventional CT [11], but no data exist regarding dual-energy/spectral image reconstructions. Some studies recommend various low- or mid-energy VMI for CCTA [12–14], but to our knowledge, segmentation reliability for vessel lumen area quantification has not been evaluated yet. VMI have different contrast and noise characteristics than conventional CT images; since spatial resolution strongly depends on contrast and noise [15, 16], increased image noise may be a concern for vessel lumen quantification tasks.

In the absence of previous demonstration, we used quantitative metrics to evaluate the impact of VMI on the accuracy and precision of coronary lumen segmentation in a high-precision phantom. Secondly, we aimed to assess the image quality of reduced-CM-dose dual-energy coronary CCTA compared with conventional CT obtained with regular CM delivery rate and volume in a large patient cohort.

## Materials and methods

### Study design

This study consisted of a phantom experiment simulating coronary arteries with high-precision vessel lumen areas and clinical research, including patients referred for CCTA. The phantom analyses were performed in an academic cardiovascular imaging laboratory (Lausanne University Hospital and Center for Biomedical Imaging, Switzerland). The clinical work was a single-center retrospective observational study approved by the local ethics committee, performed in an expert tertiary center (Hôpital Louis Pradel, Hospices Civils de Lyon, France), in which patients who received regular (range 50–90 mL) CM delivery rate and volume were assigned to group A, and those who had a reduced-CM-dose protocol (range 30–40 mL) constituted group B. Supplementary Figure A presents the study flow diagram.

### Phantom study

A previously described custom-designed resolution module [17] inserted in the center of an anthropomorphic chest phantom (QRM) simulating a normal (70 kg) or an overweight (120 kg) adult patient was used (Supplementary Figure B). The module consisted of a 2-cm-thick polymethyl methacrylate (PMMA) slab drilled with 110 holes whose diameters matched human coronary arteries, ranging from 3.00 ± 0.004 to 3.42 ± 0.004 mm [18], in 22 steps of 0.02 mm, repeated five times at different locations in the PMMA slab. The choice of PMMA was related to the ultra-high drilling precision required to serve as a reliable ground truth. The holes were homogeneously filled with two different iodinated CM concentrations (Iomeron 400, Bracco) mixed with saline. To build an experimental model recreating the lumen enhancement observed in vivo as closely as possible, we prepared Iomeprol 400/saline mixtures that exhibit similar CT numbers (at 120 kVp) as patients had in vivo (i.e., ~ 400 HU in group A, and ~ 242 HU in group B). This has brought us to use 18.5 mg I/mL to simulate regular injection and 10.5 mg I/mL to simulate a reduced CM protocol. These concentrations were adjusted to simulate lumen-to-epicardial fat contrast obtained in patients from group A (18.5 mg/mL, 400 HU) and group B (10.5 mg/mL, 242 HU). The phantom was scanned using the same parameters as in the clinical study, and the resulting DICOM images were automatically processed using a Matlab routine (MathWorks). This routine automatically segmented the vessel lumen area based on the full-width at half maximum method to outline the lumen interface and compute vessel area [19]. The difference between the known and measured vessel area was assessed on single-energy/conventional CT images (18.5 mg/mL) and 40–130-keV VMI (10.5 mg/mL), in 15 keV increments to derive segmentation accuracy and precision. The segmentation accuracy was defined as the mean area’s difference from the ground truth (drilled area), whereas the segmentation precision was defined as the standard deviation of the area measurements [19].

### Clinical study

We enrolled 203 consecutive patients referred for clinically indicated CCTA from December 2017 to August 2018. Exclusion criteria were as follows: age < 18 years, known severe allergy to iodinated CM, renal insufficiency with eGFR < 30 mL/min, coronary artery calcium score > 400. Patient characteristics and univariate comparisons between groups A and B are detailed in Table 1.

**Table 1:** Patient characteristics of the study population and univariate comparisons

	Overall	Group A, 5 mL/s	Group B, 2.5 mL/s	*p* value
*n*	203	103	100	
Age [year]	53 [23] (18–87)	50 [21.5] (18–86)	54.5 [22] (18–87)	0.362
Female sex	87 (42.8)	44 (42.7)	42 (42.0)	0.967
Height [cm]	169.3 ± 15.2 (146-198)	169.8 ± 9.1 (150-192)	168.7 ± 19.6 (146-198)	0.445
Weight [kg]	73 [21.3] (41–164)	73.5 [23.8] (47–112)	74 [18] (41–124)	0.653
Body mass index [kg/m^2^]	25 [5.7]	25 [6.1]	25.5 [5.8]	0.863
Obesity (BMI > 30 kg/m^2^)	36 (17.6)	19 (18.3)	17 (17.0)	0.957
Current or past smoking	63 (31)	37 (35.9)	26 (26.0)	0.184
Hypertension	53 (26.1)	20 (19.4)	33 (33.0)	0.037
Diabetes	21 (10.3)	10 (9.7)	11 (11.0)	0.924
Dyslipidemia	38 (18.7)	19 (18.5)	19 (19.0)	0.581
Family history of CAD	46 (22.7)	18 (17.5)	28 (28.0)	0.097

Data are means ± standard deviation or median [IQR], and (range)

*BMI* body mass index, *CAD* coronary artery disease

### Contrast medium injection protocol

Iomeprol 400 mg I/mL (Iomeron®, Bracco) was the only CM used, warmed beforehand, and injected through an 18-G catheter using a dual-head power injector. The best venous catheter insertion site was used, ideally in the right antecubital fossa. Depending on the quality of venous access (catheter location and saline test injection), the radiologist determined the CM injection rate according to the American College of Radiology Manual On Contrast Media’s recommendations [4]. The total injected CM volume was individualized based on patient weight. The injection protocols were as follows: group A (*n* = 103): volume, 1 mL/kg (maximum 90 mL); iodine delivery rate, 2 g/s; flow rate, 5 mL/s; group B (*n* = 100): volume, 0.5 mL/kg (maximum 45 mL); iodine delivery rate, 1 g/s; flow rate, 2.5 mL/s. The injection duration was 18 s in both groups and followed by a 20 mL saline flush.

### Coronary CT angiography protocols and image reconstruction

All examinations were performed on a dual-layer spectral detector CT system (IQon, Philips Healthcare), with patients lying supine, arms above the head, in a single breath-hold. If necessary, patients received intravenous beta-blockers to achieve a pre-scan heart rate no higher than 65 bpm. Helical mode CCTA with retrospective ECG-gating was performed. Detailed CCTA parameters were as follows: tube potential, 120 kVp; tube load, maximum 220 mAs; gantry revolution time, 0.27 s; automatic exposure control (angular and longitudinal), combined xyz-axis; beam collimation geometry, 64 × 0.625 mm. Bolus tracking was used, with a region of interest (ROI) placed in the descending aorta, and acquisition was triggered when an attenuation threshold of 130 HU was reached. The occurrence of allergic reactions was recorded. Volume CT dose indexes and dose-length products were retrieved from the radiation-dose structured reports. Conventional- and spectral-based images were reconstructed using a standard kernel, iterative reconstruction (iDose 3, Philips Healthcare), and section thickness of 0.9 mm. Images were reviewed offline utilizing the manufacturer’s workstation (IntelliSpace Portal 10.0, Philips Healthcare).

### Quantitative image analysis

A cardiothoracic radiologist (D.C.R.) with 7 years of experience performed all measurements. In group A, only conventional CCTA images were analyzed, whereas in group B, measurements were performed on VMI reconstructions ranging between 40 and 130 keV in 15 keV increments. Circular ROIs spanning at least 2 mm^2^ were drawn in the proximal and distal segments of the right coronary artery (RCA), left main (LM), left anterior descending (LAD), and left circumflex (LCx), to measure lumen attenuation, and special care was taken to avoid any partial volume effect. These measurements served to identify the VMI energy level providing the closest attenuation to conventional CT images. Likewise, circular ROIs of at least 2 cm^2^ were drawn in homogeneous areas of the ascending aorta, epicardial fat adjacent to the proximal RCA, trabecular bone in the center of a vertebra chosen in the mid-thoracic region, and adjacent paravertebral muscles. We selected a 2-cm^2^ area to keep the noise-dependency of values to a minimum. Contrast-to-noise ratios (CNR) were calculated using the following formula:

$$ \mathrm{CNR}=\frac{\mid \mathrm{mean}\ \mathrm{CT}\ {\mathrm{number}}_{\mathrm{lumen}}-\mathrm{mean}\ \mathrm{CT}\ {\mathrm{number}}_{\mathrm{tissue}}\mid }{\sqrt{\frac{1}{2}\left({\mathrm{SD}}_{\mathrm{lumen}}^2+{\mathrm{SD}}_{\mathrm{tissue}}^2\right)}} $$

CNR was calculated between the lumen and various tissues to approximate several physiological or pathological clinical scenarios: CNR between the lumen and fat to assess the vessel in its normal environment, between lumen and muscle to approximate a non-calcified plaque, and between lumen and trabecular bone to approximate a calcified plaque. In the phantom, CNR between the vessel and surrounding background material was computed.

### Qualitative image analysis

Two radiologists (P.C.D. and S.S-M.), with 25 and 7 years of experience in cardiovascular imaging, respectively, independently analyzed all CCTA images and could choose the best temporal phase in the cardiac cycle and the optimal grayscale windowing. Both were fully blinded to the CM injection protocol (patient groups) and clinical characteristics. In group A, the analysis was performed on conventional/single-energy images solely, whereas in group B the VMI exhibiting the closest lumen attenuation compared with group A were used. Inter-rater agreement was calculated based on the first 100 patients. The 18 coronary segments of the Society of Cardiovascular Computed Tomography model were analyzed using a 4-point Likert scale as previously published (9): excellent (no artifacts; score = 4), good (minor artifacts, good diagnostic quality; score = 3), adequate (moderate artifacts, acceptable for routine clinical diagnosis; score = 2), or poor (severe artifacts impairing accurate evaluation, segment classified as non-evaluable; score = 1). A segment was deemed assessable if scored ≥ 2, and each non-assessable segment was categorized as insufficiently enhanced or artifact-related (motion or streak). Arteries with a diameter smaller than 1.5 mm were excluded from the analysis.

### Statistical analysis

Sample size was calculated for 80% power and a type-one error rate of 5%, based on a noninferiority margin of 10% [20, 21]. To meet these requirements, we had to enroll at least 182 patients (91 in each group), assuming a 92% rate of patients with a diagnostic CCTA [21]. Results were expressed as number of subjects (percentage), as mean (± SD), or median (IQR) for non-normally distributed data, unless otherwise specified. Bivariate statistical analysis was conducted using chi-squared, Wilcoxon two-sample, or Student’s *t* test where appropriate. Variables with multiple levels were compared using the Kruskal-Wallis test with post hoc testing, and *p* values were adjusted using the Holm method. Interobserver agreement for qualitative ratings was evaluated using weighted kappa coefficients, and interpreted as follows: ≤ 0, poor; 0.01–0.20, slight; 0.21–0.40, fair; 0.41–0.60, moderate; 0.61–0.80, substantial; ≥ 0.81, excellent. *p* values < 0.05 were considered statistically significant.

## Results

### Phantom study

**Fig. 1:**
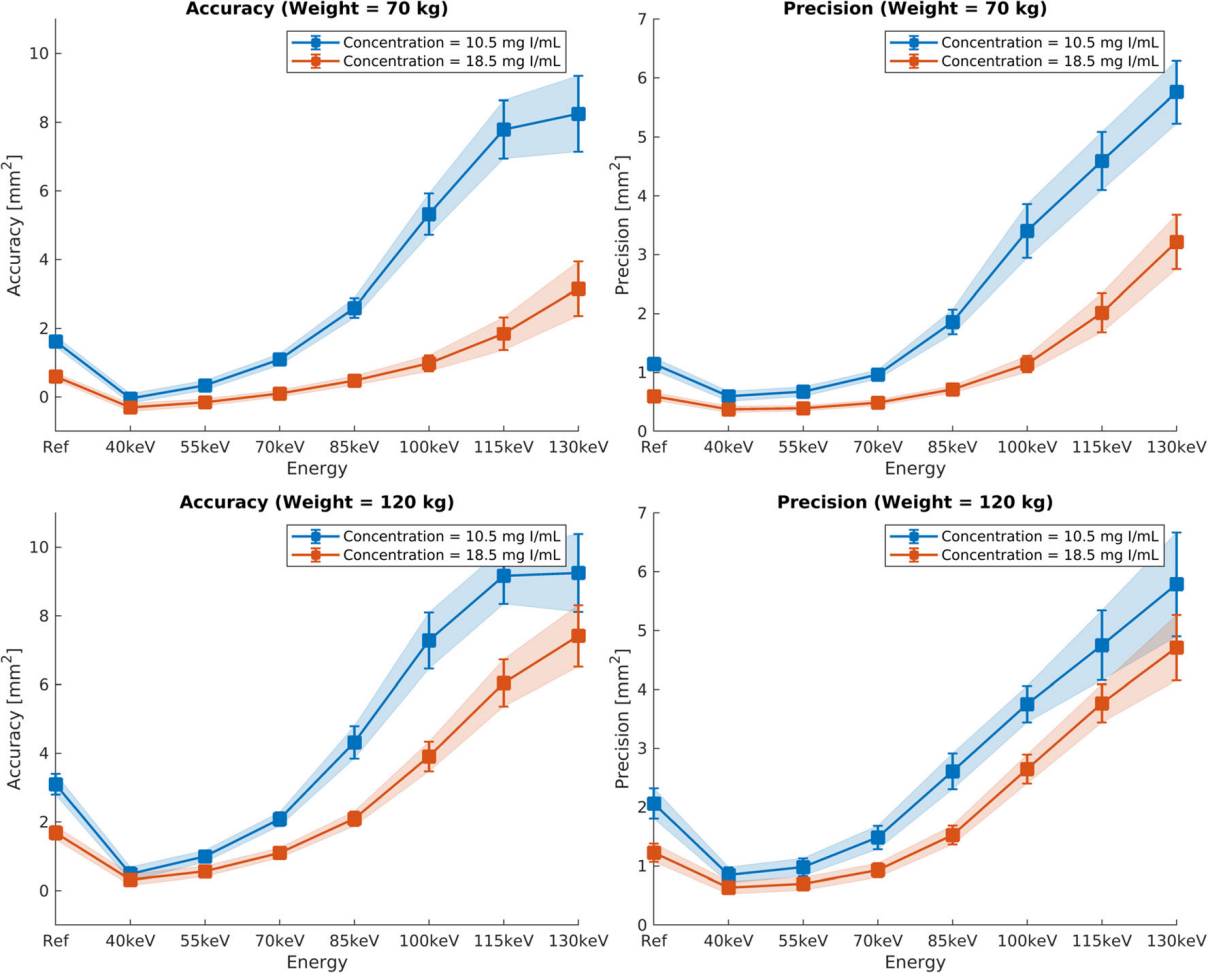
In vitro accuracy and precision of vessel lumen segmentation as a function of iodine dose (10.5 or 18.5 mg/mL) and patient size (70 or 120 kg). The conventional reconstruction “Ref” at 18.5 mL approximates protocol A in the clinical study, whereas the virtual monochromatic images (VMI) at 10.5 mg/mL approximate protocol B. Whatever the scanning condition, low VMI energies, up to a maximum of 55 keV, yielded peak segmentation accuracy and precision

For both iodine doses (18.5 mg/L and 10.5 mg/L), the algorithm achieved visually adequate segmentation of cross-sectional areas on conventional images and VMI up to 85 keV (Supplementary Figures C and D). The 55-keV VMI reconstructions (at 10.5 mg/L iodine) approximating group B patients performed better than the conventional images (at 18.5 mg/L iodine) mimicking group A patients, for accuracy (0.601 mm^2^ vs. 0.342 mm^2^, respectively, *p* < 0.001), but not for precision (0.597 mm^2^ vs. 0.675 mm^2^, respectively, *p* < 0.002), for simulated 70 kg patient size. For simulated 120 kg patient size, low-iodine 55-keV VMI provided better segmentation both regarding accuracy (0.998 vs. 1.684 mm^2^, respectively, *p* < 0.001) and precision (0.982 vs. 1.229 mm^2^, respectively, *p* < 0.001). Regardless of the patient size and CM concentration, the highest segmentation precision and accuracy were achieved at lower VMI energy levels (i.e., 40 and 55 keV), as shown in Fig. 1. The favorable effect of VMI reconstructions in reduced-CM-dose dual-energy CCTA was even larger with simulated 120 kg patient size. Besides deteriorating the segmentation precision and accuracy themselves, higher VMI reconstruction energy levels also increased the measurement variability as indicated by the error bars in Fig. 1. On the one hand, CNR between vessels and surrounding background material indicated that low-iodine 55-keV VMI preserve or improve (depending on phantom size) the CNR compared with regular-iodine conventional images and, on the other hand, suggested substantial noise reduction on VMI (Supplementary Figure E).

### Clinical study

Thirty-two (13.6%) patients were excluded due to a calcium score > 400. In the 203 patients included in the analysis, there were no significant differences in age, sex, or patient size between groups A and B (all *p* ≥ 0.36; Table 1). Patients in group B more often had hypertension (*p* = 0.03); otherwise, there were no differences regarding risk factors between groups. Examination-related characteristics are detailed in Table 2. In group B, the venous catheter was more often positioned in the left upper limb (*p* = 0.03) and more often in the forearm or wrist (*p* < 0.001). Injected CM volume was significantly higher in protocol A than in B (70.0 (IQR 8.8) mL and 40.0 (IQR 10.0) mL, respectively; *p* < 0.001). We observed no extravasation and mild allergic reaction occurred in 3/203 patients, with no significant difference between groups.

**Table 2:** CT examination characteristics and univariate comparisons

	Group A, 5 mL/s (*n* = 103)	Group B, 2.5 mL/s (*n* = 100)	*p* value
Venous catheter side, *n* (%)			
Right	86 (83.5%)	70 (70%)	0.031
Left	17 (16.5%)	30 (30%)	
Catheter location, *n* (%)			
Antecubital fossa	89 (86.4%)	64 (64%)	< 0.001
Forearm	12 (11.6%)	19 (19%)	
Hand or wrist	2 (1.9%)	17 (17%)	
Heart rate before injection [bpm]	67 [16.5]	69 [14.5]	0.617
Heart rate during injection [bpm]	63 [14]	66 [13]	0.043
Contrast medium volume [mL]	70 [8.8]	40 [10]	< 0.001
Contrast medium extravasation	None	None	
Allergy (%)			
Mild	1 (0.9%)	2 (1.8%)	0.973
Moderate	0	0	
Severe	0	0	
CTDI_vol_	23 [13.5]	21.6 [13.6]	0.540
DLP	430.7 [266.1]	392.8 [251.7]	0.620

Data are medians [IQR], or numbers (percentage). *Bpm* beats per minute, *CTDI*_*vol*_ volume computed tomography dose index, *DLP* dose-length product

### Quantitative image analysis

**Fig. 2:**
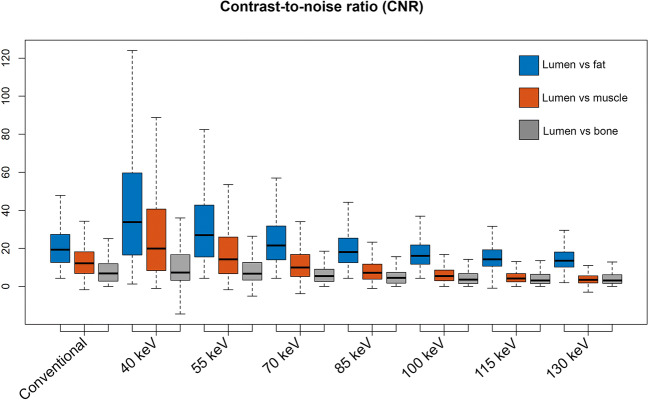
Boxplots show contrast-to-noise ratios (CNR) for different scenarios: lumen vs. fat to approximate an epicardial vessel surrounded by fat, lumen vs. muscle to approximate a non-calcified plaque, and lumen vs. trabecular bone to approximate a calcified plaque. When comparing group A (5 mL/s) with group B (2.5 mL/s reconstructed at 55 keV), no significant difference in lumen to bone CNR was found, but group B had significantly better lumen to fat and lumen to muscle CNR

The 55-keV VMI provided the closest attenuation compared with conventional reconstructions and were used for the qualitative analysis. Mean coronary lumen attenuation was significantly higher in protocol A (conventional image reconstruction) versus in B (55-keV VMI) (397.4 (IQR 131.4) HU and 380.1 (IQR 136.4) HU, respectively; *p* = 0.019). Detailed per-segment and tissue attenuation values are presented in Table 3. The attenuation in all vessel segments and tissues (fat, muscle, and bone) was significantly influenced by the VMI reconstruction energy (all *p* < 0.001). CNR between lumen and fat was significantly lower in protocol A versus in B (19.3 (IQR 11.6) and 24.9 (IQR 19.7), respectively; *p* < 0.001). CNR between lumen and muscle was significantly lower in protocol A versus in B (12.2 (IQR 8.5) and 14.3 (IQR 12.4), respectively; *p* < 0.001). Finally, CNR between lumen and bone was maintained with dual-energy CCTA (6.8 (IQR 7.3) (protocol A) and 6.7 (IQR 8.9) (protocol B), respectively; *p* = 0.423). A graphical representation (Fig. 2) across all energy levels in group B shows that reduced-CM-dose VMI reconstructions up to a maximum of 55 keV improve CNR compared with conventional CCTA (*p* = 0.012, < 0.001, and < 0.001 for lumen vs. fat, muscle, and bone, respectively).

**Table 3:** CT numbers in HU of the vascular lumen, epicardial fat, and muscle

	Reconstruction								*p* value
	Conventional	40 keV	55 keV	70 keV	85 keV	100 keV	115 keV	130 keV	
CM flow rate [mL/s]	5	2.5	2.5	2.5	2.5	2.5	2.5	2.5	
Proximal RCA	425 [116.9]	743.5 [262]	413.4 [130.6]	258.7 [80.4]	188.2 [57.6]	143.5 [45.2]	118.1 [38.2]	102 [36.8]	< 0.001
Distal RCA	414.9 [143.1]	606.3 [288.4]	346 [143.3]	222.7 [79.1]	163.9 [59.3]	130.4 [47.1]	109.4 [45.2]	95 [44]	< 0.001
LM	4451.1 [123]	828 [252]	463.5 [128.3]	284 [69.3]	205 [50.1]	160.1 [42.9]	131.1 [38.2]	114.8 [38.5]	< 0.001
Distal LAD	292.5 [135.8]	413 [244]	236.3 [134]	160 [70.9]	119.8 [55.5]	94.6 [52.6]	80 [48.6]	69.5 [45.3]	< 0.001
Distal LCx	337.9 [115.5]	496.1 [265.6]	279.6 [146.4]	185.2 [91.5]	139.8 [63.8]	116.7 [56.7]	99 [52.9]	90.7 [49]	< 0.001
Ascending aorta	466 [154.1]	871.9 [246.9]	486 [130.6]	301.4 [76.3]	211.4 [52.3]	161.4 [40.8]	130.6 [29.5]	114.2 [27.3]	< 0.001
Median	397.4 [131.4]	691.8 [259.9]	380.1 [136.4]	243.2 [77.9]	172.6 [56.4]	135.9 [47.5]	113 [42.1]	99 [40.1]	< 0.001
Epicardial fat	− 87.1 [28.6]	− 178 [47.6]	− 124 [25.3]	− 101 [19.9]	− 90 [17.3]	− 85 [16.4]	− 81.2 [17.9]	− 78.5 [15.5]	< 0.001
Muscle	103.4 [36.8]	163.1 [56.9]	108 [33.1]	81.8 [21.3]	70.5 [15.2]	63.1 [13.3]	58.4 [14.1]	56 [13]	< 0.001
Trabecular bone	201.8 [100.1]	481.6 [399.1]	297,4 [318.2]	208.5 [236.5]	169.6 [142.6]	149 [126]	138 [112.5]	130.6 [103]	< 0.001

Data are median [IQR]—univariate comparison with the Kruskal-Wallis test. IQR indicates data variability across all patients in the group

*CM* contrast medium, *keV* kilo-electronvolt, *RCA* right coronary artery, *LM* left main, *LAD* left anterior descending, *LCx* left circumflex

### Qualitative image analysis

**Fig. 3:**
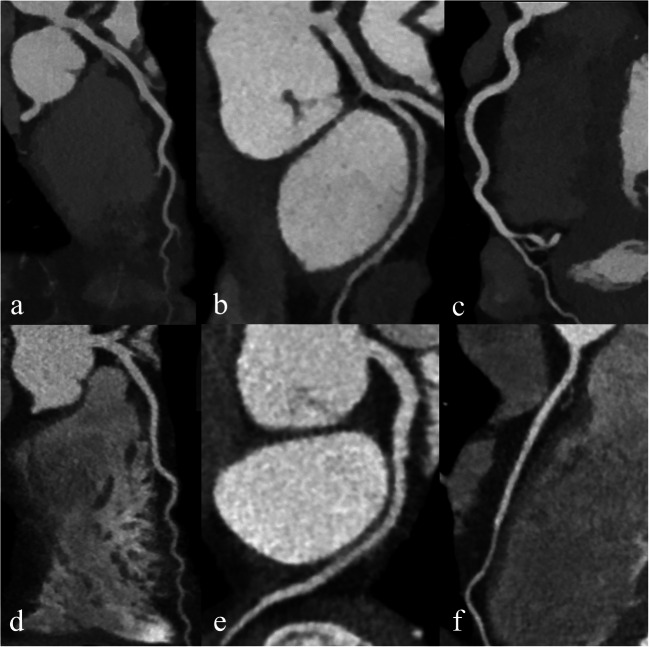
Coronary CT angiography obtained with 40 mL contrast medium injected at 2.5 mL/s and reconstructed as virtual monoenergetic images at 55 keV, depicting the LAD, LCx, and RCA (**a**–**c**), in a 68-year-old male. Coronary CT angiography obtained with 70 mL contrast agent injected at 5 mL/s and reconstructed as conventional polychromatic images, depicting the LAD, LCx, and RCA (**d**–**f**), in a 48-year-old female. LAD, left anterior descending; Cx, circumflex artery; RCA, right coronary artery; keV, kilo-electronvolt

**Fig. 4:**
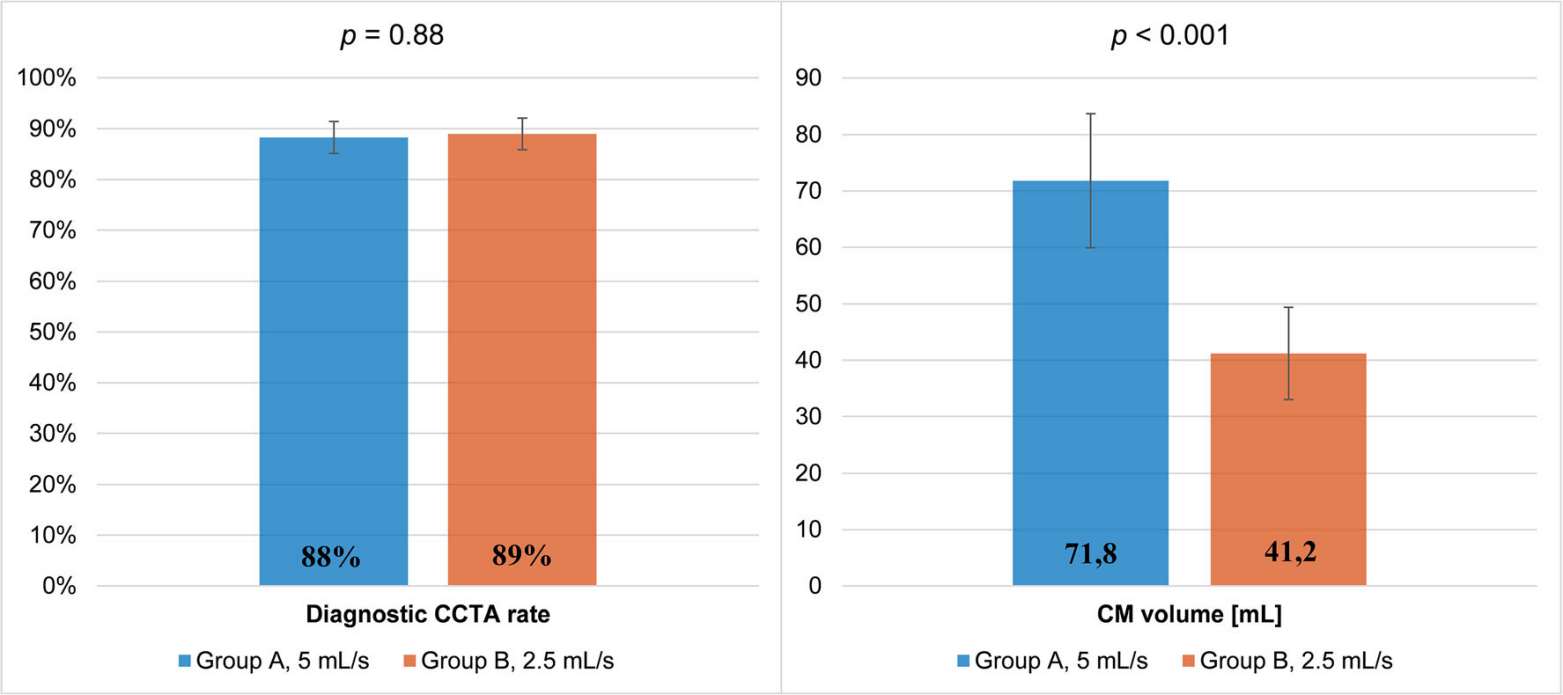
Proportion of interpretable CCTA (± standard error) and contrast agent volume (± standard deviation) in groups A and B. CM, contrast medium; CCTA, coronary computed tomography angiography. Conventional polychromatic images in group A and virtual monoenergetic images at 55 keV in group B

**Fig. 5:**
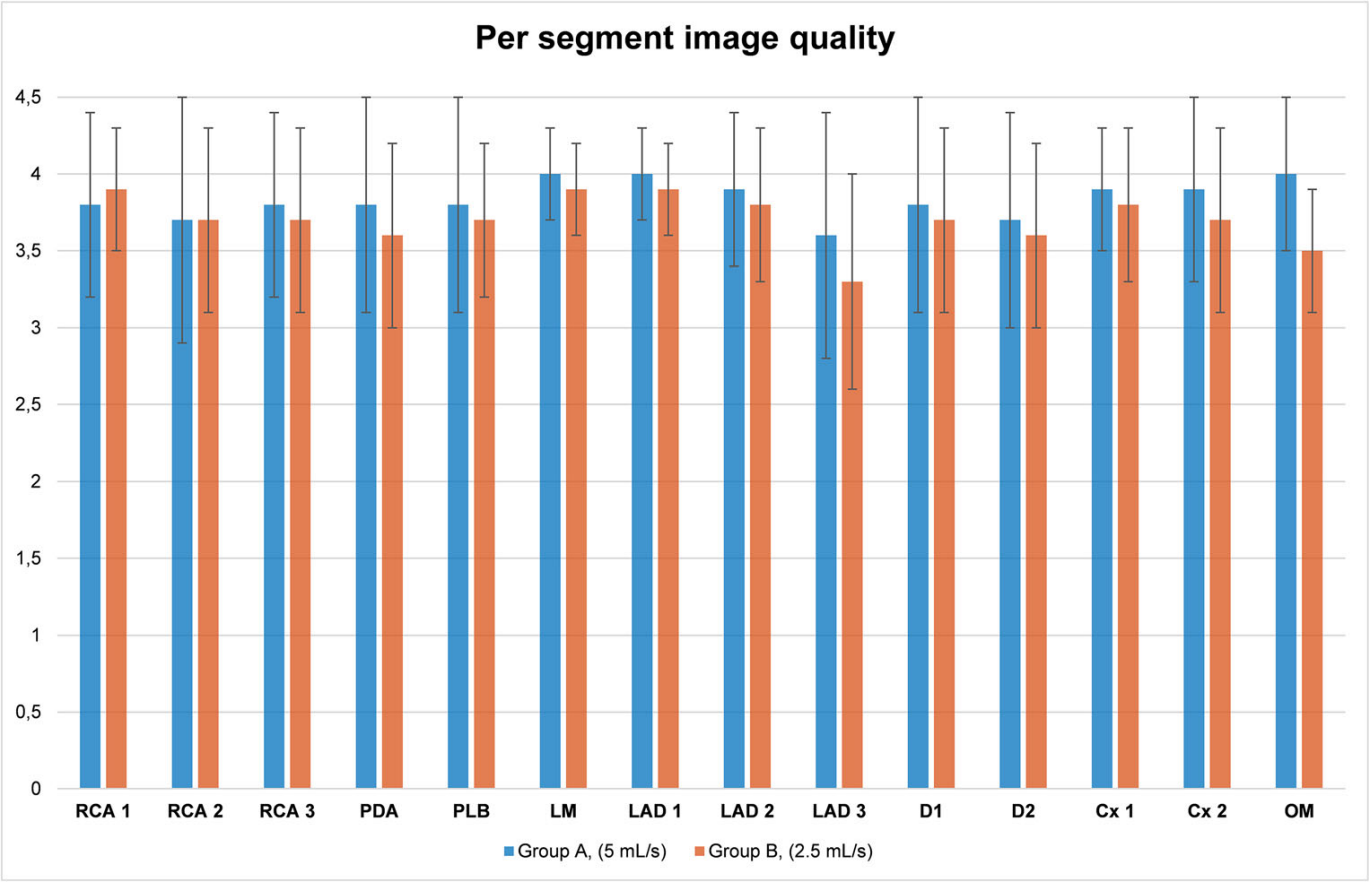
Per segment qualitative image analysis in group A and B and univariate comparison. Error bars represent standard deviation. Proximal right coronary artery (RCA 1), *p* = 0.96; mid right coronary artery (RCA 2), *p* = 0.552; distal right coronary artery (RCA 3), *p* = 0.216; posterior descending artery (PDA), *p* = 0.001; posterolateral branch (PLB), *p* = 0.010; left main (LM), *p* = 0.05; proximal left anterior descending (LAD 1), *p* = 0.004; mid left anterior descending (LAD 2), *p* = 0.023; distal left anterior descending (LAD 3), *p* = 0.001; first diagonal (D1), *p* = 0.031; second diagonal (D2), *p* = 0.059, proximal circumflex (Cx 1), *p* = 0.009; distal circumflex (Cx 2), *p* < 0.001; obtuse marginal (OM), *p* = 0.003. LAD, left anterior descending; LCx, left cirumflex; LM, left main; OM, obtuse marginal; PDA, posterior descending artery; PLB, posterolateral branch; RCA, right coronary artery

Examples of CCTA in two patients from groups A and B are illustrated in Fig. 3. While the image quality is excellent with protocol B, note that the distal LAD has slightly less lumen attenuation in protocol B than in A. Figure 4 and Table 4 summarize the results of the qualitative image analysis. The overall success rate was 88.4% (*n* = 91/103) in group A and 89% (*n* = 89/100) in group B (*p* = 0.884). Noninferiority of protocol B compared to A was inferred (95% CI of the difference = − 0.0937 to 0.0807), with a prespecified noninferiority margin of 10%. Among the segments deemed non-diagnostic, 80% (44/55) in group A and 74.6% (47/63) in group B had motion artifacts (*p* = 0.47). The inter-rater agreement was substantial (*κ* = 0.627 and 0.755 in groups A and B, respectively). Per segment image quality analysis (Fig. 5) showed that segments in group A had a slightly higher mean score than in group B, except for the RCA and D2, where differences were not statistically significant. Nevertheless, the mean scores were between good (score 3) and excellent (score 4) in all segments and groups.

**Table 4:** Qualitative analysis

		Group A, 5 mL/s	Group B, 2.5 mL/s	*p* value
*n* patients		103	100	
Patients with diagnostic CCTA, *n* (%)		91/103 (88.4%)	89/100 (89%)	0.884
Patients with non-diagnostic CCTA, *n* (%)		12/103 (11.6%)	11/100 (11%)	
	Non-diagnostic due to motion artifacts	10/12 (83.3%)	10/11 (90.9%)	0.29
	Non-diagnostic due to inadequate enhancement	2/12 (16.7%)	1/11 (9.1%)	
Total segments analyzed		1441	1310	
Image quality score				
Excellent, *n* (%)		1220/1441 (84.7%)	949/1310 (72.4%)	
Good, *n* (%)		74/1441 (5.1%)	267/1310 (20.4%)	
Adequate 3, *n* (%)		35/1441 (2.4%)	31/1310 (2.4%)	
Poor (non-evaluable), *n* (%)		55/1441 (3.8%)	63/1310 (4.8%)	
	Poor due to motion artifacts	44/55 (80%)	47/63 (74.6%)	0.486
	Poor due to inadequate enhancement	11/55 (20%)	16/63 (25.4%)	
Average image quality score per segment (± SD)		3.9 (± 0.4)	3.7 (± 0.5)	< 0.001

Data are numbers with percentages in parentheses or mean ± standard deviation. *CCTA* coronary computed tomography angiography

## Discussion

Our study aimed to compare conventional CCTA with reduced-CM-dose dual-energy CCTA in both a resolution phantom and large patient cohort; the reduced-iodine-dose spectral CT protocol outperformed conventional CT in vitro and was noninferior in vivo. This is the largest study assessing the potential to reduce CM with dual-energy CCTA, and the only one using an appropriate noninferiority statistical analysis.

Today, CCTA is used in a broad range of clinical applications, even in high-risk coronary artery disease [22]. That is, upon plaque detection, quantification is required to guide patient management. Stenosis quantification must be precise, whether through semi-automatic stenosis assessment by a radiologist or with automated fractional flow reserve CT (FFR-CT) [23]. Both methods rely on an accurate representation of the vessel’s cross-sectional area, which led us to conduct a phantom study to evaluate cross-sectional segmentation accuracy. Not only did VMI help preserve the segmentation accuracy and precision with reduced CM, but VMI even improved segmentation in the phantom setup simulating larger patients. CCTA in obese patients remains challenging and usually results in a higher radiation dose [24]. Contrary to low-tube-voltage scanning (70–80 kVp), dual-energy CCTA with VMI allowed CM reduction even in patients > 25 kg/m^2^ [25], since our study included patients with BMI up to 40 kg/m^2^. The optimal VMI energy level for image analysis is still debated and may depend upon the system used; in the early days of DECT, low-keV VMI had higher noise, and initial reports advocated VMI in the range of 65–80 keV [12, 26]. Nevertheless, much has already been achieved for making low-energy VMI suitable for clinical use, and more recent studies emphasize the value of reconstructions at lower energies [14, 27].

In the patient study, we found a minimal compromise regarding qualitative image quality, which agrees with Raju et al [7]. Specifically, the distal coronary segments received slightly lower image quality scores in group B. Nevertheless, the mean image quality scores were between good and excellent in both study groups, and CM reduction did not impair the diagnosis. We calculated CNR between the lumen and various densities approximating calcified, non-calcified, and lipid-rich plaques to evaluate whether the detectability of different plaque types was maintained on low-iodine CCTA images. While such CNR measurements do not directly relate to calcified or non-calcified coronary atherosclerotic plaques, the analyses account for the specific spectral behavior of fat, soft-tissue, and calcium-containing materials to show trends that can be expected when using VMI. The CNR between the lumen and calcium was comparable in groups A and B, despite a lower lumen-to-bone contrast on VMI, which is attributed to the lower noise present on spectral-based images. VMI and other dual-layer CT-derived spectral reconstructions use noise anti-correlation to reduce the overall noise magnitude [28]. On the other hand, the CNR between the lumen and fat was significantly higher in group B, mostly because fat exhibits lower attenuation at 55 keV (− 122 HU in our study) than on conventional images (− 88 HU), which confirms early findings by Oda et al [8]. This indicates that plaque composition analysis may be improved with reduced CM and lower energy VMI, but also highlights the potential usefulness of lower energy VMI reconstructions for plaque composition analysis, especially the detection of lipid-rich core, a clinically relevant determinant of plaque vulnerability [29].

To achieve proper arterial enhancement, current guidelines recommend the use of high contrast medium (CM) injection rates and doses, typically on the order of 5–7 mL/s, at a concentration of 270–400 mg I/mL, and a total volume of 50–120 mL [30]. Such injection parameters ideally involve 18-G venous catheters located in the right antecubital fossa. Nevertheless, we found that almost a quarter of patients had their catheter placed in the forearm or the wrist, which is reflective of the challenges faced in daily patient management. Patients with a catheter inserted in the forearm or the wrist were almost three times more likely to be subjected to the group with lower CM delivery rate, indicating that protocol B may have helped manage patients with poor vein integrity.

Despite careful design, our study has several limitations. First, we could not reliably assess the accuracy of stenosis quantification in vivo because of the low incidence of coronary artery disease in our study population; less than 10% of patients had a coronary artery stenosis ≥ 70%. Evaluation of diagnostic accuracy should be considered in another patient population for whom an indication for invasive coronary angiography exists. However, the phantom study provides encouraging results in that field. Second, we used retrospective ECG-gating resulting in relatively high radiation dose delivery. This choice was linked to *z*-axis coverage of the dual-layer spectral detector, which is 4 cm; still, a new platform with 8 cm detector coverage is in the process of being released, and wide-area (16 cm) detectors are expected to become available for spectral ECG-gated cardiac CT. Third, we did not test pre- and post-injection serum creatinine levels, nor did we include at-risk patients (with an estimated glomerular filtration rate < 30 mL/min); therefore, we cannot comment on the impact of CM on kidney function. Fourth, the injection protocol was chosen individually by the radiologist in charge; however, since patients with poor vein integrity more often received reduced-CM-dose dual-energy CCTA, we do not expect a relevant bias towards too good image quality in this group. Finally, our results might not apply to other DECT systems since ECG-gated DECT scanning is not available on all DECT platforms and because certain vendors need to cut back temporal resolution when using spectral mode, some of which may result in increased radiation dose, motion, or misalignment artifacts. Despite this, when dual-source CT is used in spectral mode, its temporal resolution is still similar to that of dual-layer CT, and that fast kVp switching platforms will soon support faster gantry rotation speed, which may compensate for the loss of temporal resolution.

In conclusion, dual-energy CCTA allows for a 40% iodine dose and 50% injection rate reduction while preserving diagnostic image quality and may facilitate the management of patients with poor venous access. Furthermore, low-keV VMI improve the CNR between lumen versus fat and muscle, offering an opportunity to enhance the contrast between various plaque components, which is key for the quantification of the lipid-rich core. In vitro results indicate that low-keV VMI improve vessel area quantification, especially in simulated overweight subjects.

## Supplementary information

ESM 1 (DOCX 1407 kb)

## Abbreviations

CCTA: Coronary computed tomography angiography
CM: Contrast medium
CNR: Contrast-to-noise ratio
LAD: Left anterior descending
LCx: Left circumflex
LM: Left main
RCA: Right coronary artery
ROI: Region of interest
VMI: Virtual monochromatic image

## Declarations

### Guarantor

The scientific guarantor of this publication is Prof. Philippe C. Douek, MD, PhD.

### Statistics and biometry

One of the authors has significant statistical expertise.

### Methodology

• retrospective

• observational

• multicenter study
