# Supplementary material for: Reduced-iodine-dose dual-energy coronary CT angiography: qualitative and quantitative comparison between virtual monochromatic and polychromatic CT images
Source: Eur Radiol. 2021 Mar 19;31(9):7132–42. doi: 10.1007/s00330-021-07809-w (PMC8379124; doi:10.1007/s00330-021-07809-w)

**Supplementary material**

**Supplementary Figure A**. Flow diagram of phantom and clinical studies.


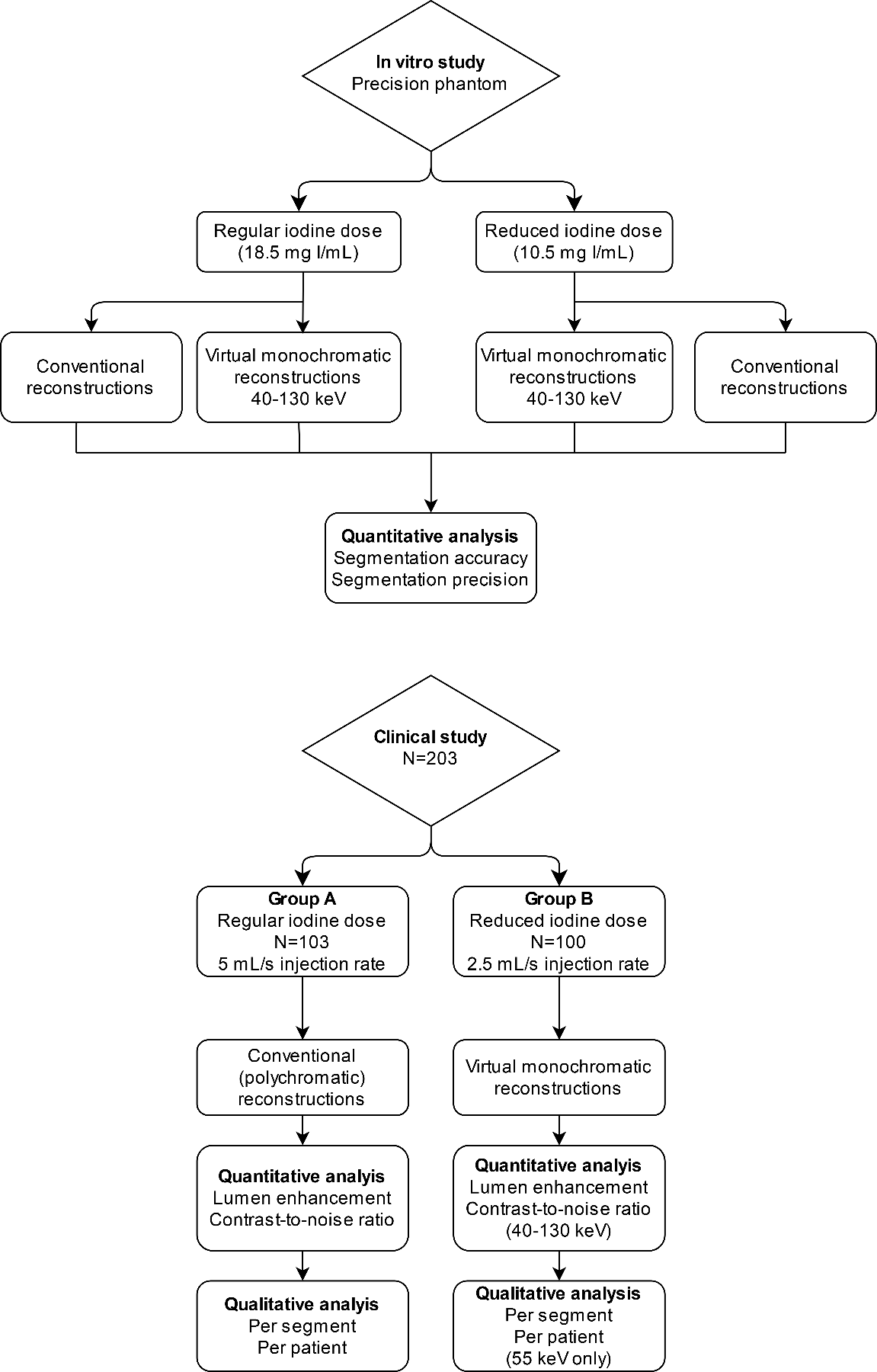


**Supplementary Figure B**. Phantom setup. (**a**) Schematic representation of the drilling layout of the central polymethyl methacrylate resolution module. In total, the 110 drilled holes consisted of 22 different diameters ranging from 3.00 to 3.42 mm, in continuous steps of 0.02 mm, each repeated five times at varying locations in the module. (**b**) Photographs of the anthropomorphic thorax phantom (left) alone simulating a thin (~70 kg) adult subject, and (right) with the addition of a 5-cm-thick extension ring mimicking the attenuation of the subcutaneous adipose tissue in an overweight (~120 kg) patient. Panel (a) adapted with permission from Yerly et al [17].


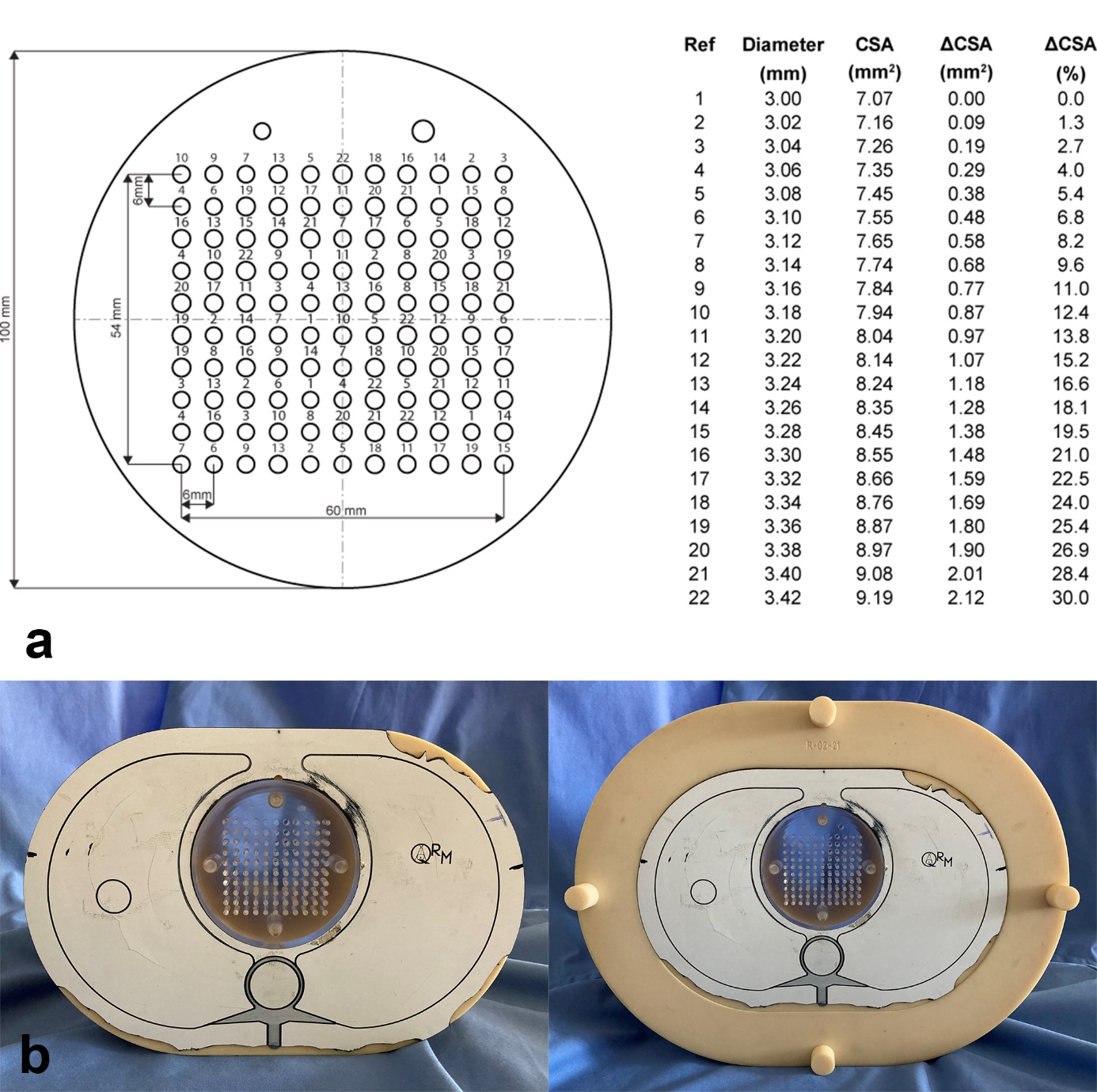


**Supplementary Figure C.** Automatic segmentation of the high-precision coronary lumen phantom using the full-width at half-maximum technique, 18.5 mg/mL iodine dose, mimicking the regular CM injection in group A. Conventional polychromatic (a), 55 keV virtual monochromatic images (VMI) (b), 85 keV VMI (c), and 115 keV VMI (d). The algorithm achieved visually adequate lumen segmentation on conventional images and across all VMI energy levels up to 85 keV; at 115 keV, however, the segmentation became incomplete and erratic.


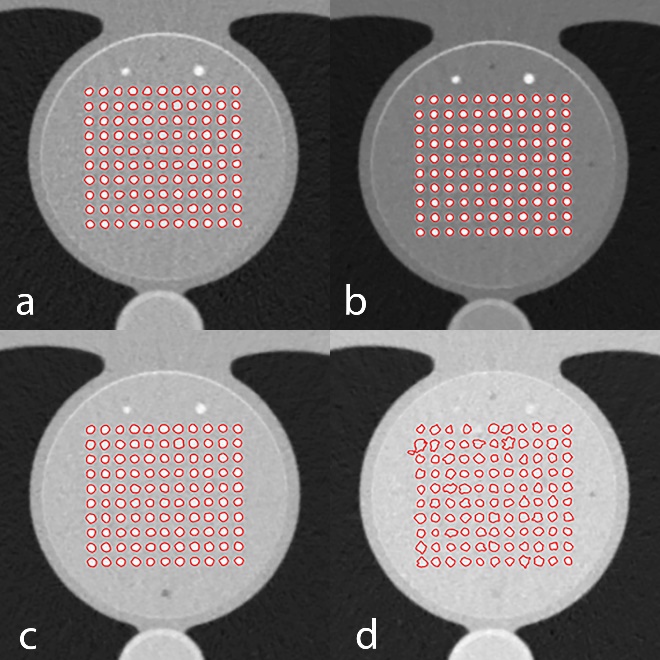


**Supplementary Figure D.** Automatic segmentation of the high-precision coronary lumen phantom using the full-width at half-maximum technique, 10.5 mg/mL iodine dose, mimicking the reduced-CM-dose injection in group B. Conventional polychromatic (a), 55 keV virtual monochromatic images (VMI) (b), 85 keV VMI (c), and 115 keV VMI (d). The algorithm achieved visually adequate lumen segmentation on conventional images and 55 keV VMI; from 85 keV and up, the segmentation performance dropped gradually.


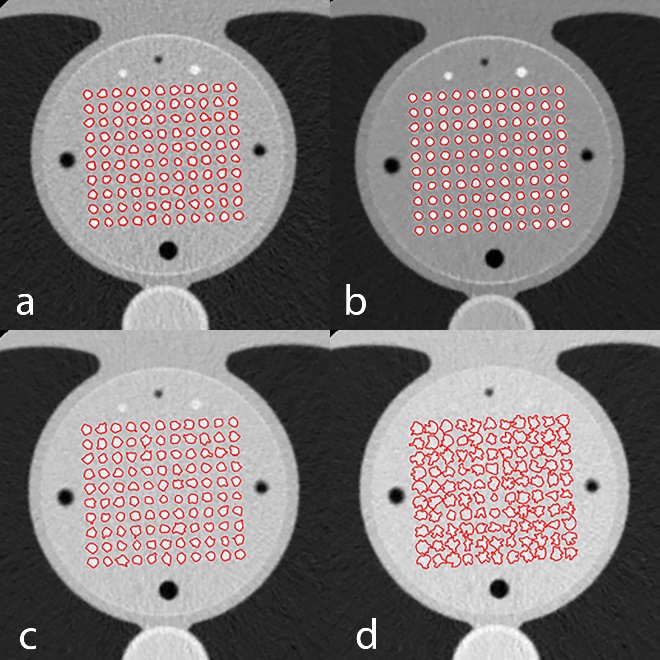


**Supplementary Figure E.** In vitro contrast-to-noise ratio (CNR) between vessel lumen and surrounding background material as a function of iodine dose (10.5 or 18.5 mg I/mL) and simulated patient size (70 or 120 kg). The conventional reconstruction "Ref" at 18.5 mL approximates protocol A in the clinical study, whereas the virtual monochromatic images (VMI) at 10.5 mg/mL approximate protocol B. Low-iodine (10.5 mg I/mL) 55 keV VMI preserved the CNR compared with regular-iodine conventional "Ref" reconstructions at normal phantom size and provided markedly better CNR with the overweight phantom size. Whatever the scanning condition, the lower the VMI energy, the higher the CNR.


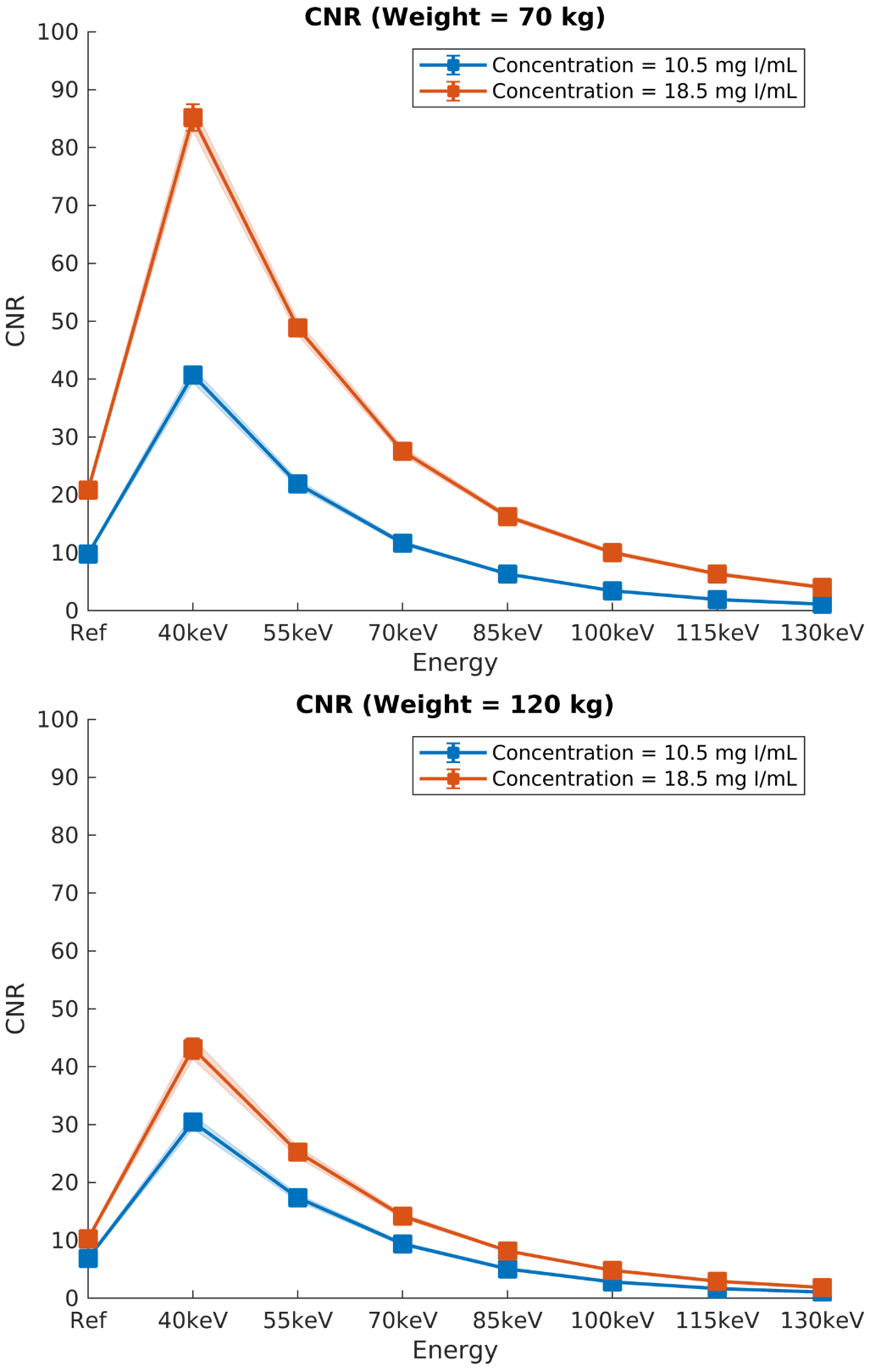

Supplement: Supplementary file 1 — (DOCX 1407 kb) [file 330_2021_7809_MOESM1_ESM.docx]
